# NATIONAL ELDER MISTREATMENT STUDY WAVE II: MENTAL HEALTH CORRELATES OF FINANCIAL MISTREATMENT

**DOI:** 10.1093/geroni/igz038.692

**Published:** 2019-11-08

**Authors:** Melba Hernandez-Tejada, Ron Acierno, Jordan Watkins, Gabrielle Frook, Wendy Muzzy, Georgia Anetzberger, Mara Steedley

**Affiliations:** 1 Medical University of South Carolina, Charleston, South Carolina, United States; 2 Case Western Reserve University, Cleveland, Ohio, United States

## Abstract

Objective: Whereas prevalence of elder financial mistreatment has received increased attention over the past decade, health and mental health correlates are rarely studied. Thus, the potential relevance of financial abuse to mental health and perceived health is relatively unknown, and the objective of this article is to illustrate this relationship. Method: The second wave of the National Elder Mistreatment Study used random digit dialing telephone survey methodology to assess both recent financial mistreatment and its potential mental health correlates (i.e., diagnoses of depression, post-traumatic stress disorder [PTSD], generalized anxiety disorder [GAD], and self-ratings of physical health) in 774 older adults. Results: The study indicated that past-year Wave II financial mistreatment was associated with significantly increased likelihood of depression, PTSD, GAD, and poor self-rated health; and financial mistreatment perpetrated by family members was associated with particularly increased risk of depression. Discussion: Assessment of mental health is relevant and important in cases of financial abuse.

